# Selective Enrichment Yields Robust Ethene-Producing Dechlorinating Cultures from Microcosms Stalled at *cis*-Dichloroethene

**DOI:** 10.1371/journal.pone.0100654

**Published:** 2014-06-20

**Authors:** Anca G. Delgado, Dae-Wook Kang, Katherine G. Nelson, Devyn Fajardo-Williams, Joseph F. Miceli, Hansa Y. Done, Sudeep C. Popat, Rosa Krajmalnik-Brown

**Affiliations:** 1 Swette Center for Environmental Biotechnology, Biodesign Institute, Arizona State University, Tempe, United States of America; 2 School of Sustainable Engineering and the Built Environment, Arizona State University, Tempe, United States of America; Wageningen University, Netherlands

## Abstract

*Dehalococcoides mccartyi* strains are of particular importance for bioremediation due to their unique capability of transforming perchloroethene (PCE) and trichloroethene (TCE) to non-toxic ethene, through the intermediates *cis*-dichloroethene (*cis*-DCE) and vinyl chloride (VC). Despite the widespread environmental distribution of *Dehalococcoides*, biostimulation sometimes fails to promote dechlorination beyond *cis*-DCE. In our study, microcosms established with garden soil and mangrove sediment also stalled at *cis*-DCE, albeit *Dehalococcoides mccartyi* containing the reductive dehalogenase genes *tceA, vcrA* and *bvcA* were detected in the soil/sediment inocula. Reductive dechlorination was not promoted beyond *cis*-DCE, even after multiple biostimulation events with fermentable substrates and a lengthy incubation. However, transfers from microcosms stalled at *cis*-DCE yielded dechlorination to ethene with subsequent enrichment cultures containing up to 10^9^ *Dehalococcoides mccartyi* cells mL^−1^. *Proteobacterial* classes which dominated the soil/sediment communities became undetectable in the enrichments, and methanogenic activity drastically decreased after the transfers. We hypothesized that biostimulation of *Dehalococcoides* in the *cis*-DCE-stalled microcosms was impeded by other microbes present at higher abundances than *Dehalococcoides* and utilizing terminal electron acceptors from the soil/sediment, hence, outcompeting *Dehalococcoides* for H_2_. In support of this hypothesis, we show that garden soil and mangrove sediment microcosms bioaugmented with their respective cultures containing *Dehalococcoides* in high abundance were able to compete for H_2_ for reductive dechlorination from one biostimulation event and produced ethene with no obvious stall. Overall, our results provide an alternate explanation to consolidate conflicting observations on the ubiquity of *Dehalococcoides mccartyi* and occasional stalling of dechlorination at *cis*-DCE; thus, bringing a new perspective to better assess biological potential of different environments and to understand microbial interactions governing bioremediation.

## Introduction

*Dehalococcoides mccartyi* is a newly classified genus and species belonging to the *Dehalococcoidia* class in the phylum *Chloroflexi* [1]. The members of this genus respire halogenated compounds with an array of carbon backbones of biogenic and anthropogenic origin (i.e., ethenes [2], [3], benzenes [4], phenols [5], and biphenyls [6]). Of particular importance for bioremediation are *Dehalococcoides mccartyi* strains that reduce the widespread soil and groundwater contaminants perchloroethene (PCE), trichloroethene (TCE), and the daughter chlorinated products, *cis*-dichloroethene (*cis*-DCE) and vinyl chloride (VC) to the non-toxic, non-chlorinated end product, ethene [1]–[3], [7]. These strains couple the reductive dechlorination of these chlorinated ethenes to growth using H_2_ as electron donor and acetate as carbon source [1]. Hence, stimulation of indigenous *Dehalococcoides* (biostimulation) or addition of laboratory-cultivated consortia containing *Dehalococcoides* (bioaugmentation) are bioremediation engineering avenues utilized to decontaminate and restore sites polluted with chlorinated ethenes [8], [9]. The potential for partial reduction of PCE and TCE to *cis*-DCE extends to multiple bacterial genera [10]. On the other hand, the capacity to dechlorinate chlorinated ethenes to ethene is, to date, unique to *Dehalococcoides mccartyi*.

A range of (mostly) contaminated environments has served as microbial inocula for *Dehalococcoides* enrichment cultures throughout the two decades of research on reductive dechlorination. Table 1 contains a compilation of cultures employed in fundamental studies and in bioaugmentation research/applications for PCE or TCE dechlorination. Development of these enrichment cultures is a lengthy process [11] as the enrichments must be actively fed and transferred to maintain the desired biological activity. Careful consideration is given to any environmental sample (soil, sediment, or groundwater) before pursuing this labor- and time-intensive work. Often crucial in deciding to i) develop novel reductively dechlorinating enrichment cultures, ii) biostimulate, and iii) bioaugment a contaminated site is evidence of reductive dechlorination to VC and ethene. Hence, VC and ethene are measured either in laboratory microcosm experiments or directly, during evaluation of contaminated sites [12]–[14]. This information is not always reported *per se*; however, for the majority of the enrichment cultures in Table 1, there was evidence of desired biological activity through one or both assessment methods.

**Table 1:** Environmental inocula and enrichment conditions of chlorinated ethene-dechlorinating cultures.

Culture	Inoculum source	Contamination and/or anthropogenic activity	Enrichment	
			Chlorinated e^−^ acceptor	e^−^ donor and carbon source
Unnamed [15] ^a^	Sludge, Ithaca wastewater treatment plant, NY	Wastewater	PCE	Methanol and acetate
Pinellas [16]	Soil and groundwater, Department of Energy's Pinellas site, Largo, FL	Chlorinated solvents (mostly TCE)	TCE	Lactate
ANAS [17]	Soil, Alameda Naval Air Station, CA	Chlorinated solvents (mostly TCE) and waste oil	TCE	Lactate
KB1 [18]	Soil and groundwater, Southern Ontario contaminated site, Canada	TCE	TCE	Methanol
Unnamed [19] ^b^	Aquifer material, Bachman Road Residential Wells site, Oscoda, MI	PCE	*cis*-DCE	Lactate
Victoria [20] ^c^	Aquifer material, Victoria contaminated site, TX	PCE	PCE	Benzoate
Unnamed [21] ^d^	Sediment, Red Cedar River, MI	No contamination	PCE	H_2_ and acetate
PM [22]	Aquifer material, Point Mugu Naval Air Weapons Station, CA	TCE	TCE	Butanol
EV [22]	Groundwater, Evanite site, Corvallis, OR	TCE	PCE	Butanol
SDC-9 [23]	Aquifer material, contaminated site, Southern CA	Chlorinated solvents	PCE	Lactate
Hawaii-05 [24]	Aquifer material, Hickam Air Force Base, HI	Chlorinated solvents	TCE	Lactate
PKJS [24]	Aquifer material, Air Force Plant PJKS, CO	TCE	TCE	Lactate
DehaloR∧2 [25]	Estuarine sediment, Chesapeake Bay, MA	Wastewater effluent	TCE	Lactate and methanol
ZARA-10 (this study)	Garden soil, Cuzdrioara, Romania	No contamination	TCE	Lactate and methanol
LINA-09 (this study)	Mangrove sediment, Carolina, Puerto Rico	No contamination	TCE	Lactate and methanol
ISLA-08 (this study)	Groundwater sediment, Parris Island MCRD Site 45, SC	PCE	TCE	Lactate and methanol

Enrichment originating *Dehalococcoides mccartyi* strain ^a^195, ^b^BAV1, ^c^VS, and ^d^FL2.

Observations on the presence of *Dehalococcoides* and the stalling of PCE/TCE dechlorination at *cis*-DCE or VC are also common. This puzzling outcome has been reported in soil and sediment microcosm studies and in bench-scale bioremediation scenarios [12], [16], [25]–[28], as well as at contaminated sites undergoing bioremediation [8], [14], [29]. Whereas some of the abovementioned works did not put forth an explanation on the inability to achieve dechlorination of *cis*-DCE or VC, the most commonly proposed reason was the absence of *Dehalococcoides mccartyi* strains with DCE- and VC-respiring metabolic capabilities [8], [16], [26], [28], [30]. Nonetheless, this unpredicted outcome was also noted even when the identified *Dehalococcoides mccartyi* genes *vcrA* and *bvcA* coding for VC reductive dehalogenase enzymes were detected [27]. Yet, neither VC reduction nor increases in *Dehalococcoides mccartyi* occurred in microcosms biostimulated with a fermentable substrate as the precursor for H_2_ and acetate [27].

We hypothesize that, often, the discrepancy between the expected and the observed activities of *Dehalococcoides* is *not* due to their metabolic potential; instead, it is a consequence of the intrinsic competition for electron donor (H_2_) in soils and sediments, driven by a variety of electron acceptors such as nitrate, Fe (III), sulfate, and bicarbonate (HCO_3_^−^). Electron donor competition was recognized early on as an important phenomenon that needed to be characterized in order to predict, explain, and optimize reductive dechlorination by *Dehalococcoides* [12], [31]–[33]. This need led to a number of studies showing that donor-competing terminal electron accepting processes affect not only the rates but also the extent of reductive dechlorination of chlorinated ethenes [31], [32], [34]–[40]. At contaminated sites undergoing *in situ* bioremediation, these processes could lead to minimal biostimulation of *Dehalococcoides*, prolonged lag times before the onset of dechlorination, and/or incomplete dechlorination. But despite the acknowledgement of donor competition as a variable in reductive dechlorination, its importance is often dismissed at field sites on the grounds that fermentable substrates responsible for H_2_ production are added in excess [13].

In this study, microcosms were biostimulated with TCE and fermentable substrates to promote growth of *Dehalococcoides mccartyi* but stalled at *cis*-DCE, irrespective of the fact that electron donor was supplied ∼150 times in stoichiometric excess for complete dechlorination of TCE to ethene. Our findings strongly support electron donor competition for the inability to produce VC and ethene in microcosms stalled at *cis*-DCE. Transfers from stalled microcosms in the absence of soil/sediment produced ethene and yielded in subsequent enrichment cultures robust growth of *Dehalococcoides mccartyi* and fast rates of dechlorination.

## Materials and Methods

### Ethics Statement

No specific permits were required for the sampling activities in Cuzdrioara, Romania and Carolina, Puerto Rico. The Cuzdrioara location is privately-owned by the family of the first author (AGD). Please contact AGD for future permissions. The sampling location in Carolina is not privately-owned or protected in any way. The core sediments from Parris Island Marine Corps Recruit Depot (MCRD) Site 45 were donated by Paul C. Johnson from Arizona State University who was authorized by MCRD to collect these samples from their site. The sampling work did not involve any endangered or protected species in either location.

### Environmental Sources

The soil and sediment samples originated from the following geographic locations: Cuzdrioara, Cluj County, Romania (47.17°N, 23.92°E), Carolina, Puerto Rico, USA (18.34°N, 65.95°W), and Parris Island MCRD Site 45, Beaufort County, South Carolina, USA (32.33°N, 80.69°W). The Cuzdrioara soil was collected from an uncontaminated vegetable garden from a depth of ∼15 cm. The Carolina sediment was sampled from an uncontaminated tropical mangrove with a shallow water table (10–15 cm). The sediments from Parris Island were core samples collected from a 5 m depth in an area of the military base contaminated with PCE. A site description with sampling locations for Parris Island MCRD Site 45 was published elsewhere [41]. Once brought to the laboratory, all soils and sediments were stored at 4°C until the establishment of microcosms.

### Microcosms and Enrichment of Soil/Sediment-Free, Chlorinated Ethene-Respiring *Dehalococcoides* Cultures

We established the following microcosms: Cuzdrioara soil, n = 3; Carolina sediment, n = 3; and Parris Island sediment, n = 20 (replicates from 10 core sections evenly dispersed throughout a 5 m depth profile) in 30 mM HCO_3_^−^-buffered, reduced anaerobic mineral medium described [25]. Each microcosm consisted of 5 g soil or sediment in 160-mL glass serum bottles with 100 mL medium. The initial pH of the medium was 7.8. We added to each microcosm 0.2–0.3 mmol L^−1^ TCE (nominal concentration). Additionally, we added the fermentable substrates lactate (5 mM) and methanol (12 mM) as H_2_ and acetate precursors, 1 mL ATCC MD-VS vitamin supplement (ATCC, USA), and 50 µL of vitamin B_12_ from a 1 g L^−1^ stock solution. The microcosm bottles were incubated statically at 30°C. Cuzdrioara and Carolina microcosms were incubated for 200 days, during which time 5 mM lactate was re-added on two separate occasions (days 46 and 180).

We performed serial transfers (10% vol/vol) into same size serum bottles using the same medium compositions to remove the soil or sediment. The microcosm bottles were shaken vigorously and allowed to settle for 15 minutes so that the supernatant was mostly devoid of soil or sediment when transferred to the new bottles. Upon each transfer, we supplied additional TCE, lactate, and methanol. After three consecutive transfers, we named the enrichment culture from Cuzdrioara soil, ZARA-10, from Carolina sediment, LINA-09, and from Parris Island sediment, ISLA-08. At all times when handling these cultures, we employed aseptic technique and took precautions to avoid cross-contamination of the cultures. We maintained the three enrichment cultures by feeding them 3–4 times consecutively with 0.5 mmol L^−1^ TCE, 5 mM lactate, and 12 mM methanol or 1 mmol L^−1^ TCE, 5 mM lactate, and 24 mM methanol. Each addition of TCE was allowed to proceed to ≥80% ethene. We flushed the headspace of the bottles with ultra-high purity N_2_ gas to remove headspace gases that accumulated as a result of dechlorination (ethene and VC), fermentation (CO_2_) and methanogenesis (CH_4_) before adding a new dose of TCE and fermentable substrates. Removal of CO_2_ from the headspace would also raise the pH of the medium, which decreased as a result of dechlorination and fermentation. When not actively used in experiments, we kept stock cultures of the soil/sediment-free enrichments in a 4°C refrigerator. No significant loss of activity was observed in these cultures even after several months of storage at 4°C, consistent with published findings [40].

### ZARA-10 and LINA-09 Bioaugmentation Experiments

Bioaugmentation experiments were carried out to evaluate whether ZARA-10 and LINA-09 soil/sediment-free cultures could dechlorinate TCE to ethene in the soil and sediment from which they originated. We setup 120-mL glass serum bottles (four bottles per culture) with 2.5 g soil or sediment, 50 mL reduced anaerobic medium, 0.25 mmol L^−1^ TCE, 5 mM lactate and 12 mM methanol, and 1% inoculum vol/vol (0.5 mL) ZARA-10 or LINA-09 culture, respectively. We measured the dechlorination of TCE to ethene in time-course experiments.

### Gas and Liquid Chromatography Analyses

200 µL gas samples were extracted from the headspace of bottles to measure chlorinated ethenes, ethene, and methane using a Shimadzu GC-2010 (Columbia, USA) instrument with a flame ionization detector (FID). Details on the column type and properties, GC temperature and pressure profiles, calibration curves, and detection limits for each compound measured on the GC-FID were published elsewhere [34], [42], [43]. H_2_ was also measured from 200 µL gas samples from the headspace of the bottles using a Shimadzu GC equipped with a thermal conductivity detector (TCD). However, all samples assayed were below our GC-TCD H_2_ detection limit of 0.8% vol/vol.

1 mL liquid samples were prepared for high performance liquid chromatography (HPLC; Shimadzu LC-20AT) through filtration using a 0.2 µm polyvinylidene fluoride membrane syringe filter (Pall Corporation, USA). We measured lactate, acetate, propionate, and methanol using the method outlined by Parameswaran et al. [44], except the elution total time was 40 minutes and the column temperature was constant at 50°C.

### Microbial Community Analyses

We extracted genomic DNA from 0.25 g of soil or sediment using the PowerSoil DNA Isolation Kit (MO BIO Laboratories, Inc., USA) following the protocol recommended by the manufacturer. For soil/sediment-free consortia, we used pellets formed from 1.5 mL liquid culture. Before DNA extraction, we pretreated the pellets as noted in Ziv-El et al. [25]. Then, we followed the protocol for Gram-positive bacteria outlined in the DNeasy Blood and Tissue Kit (QIAGEN, USA).

We determined the relative community structure using 454 pyrosequencing in soils, sediments, and soil/sediment-free enrichment cultures. The DNA samples were analyzed at the Research and Testing Laboratory (Lubbock, TX). The targets were the combined V2 and V3 regions of the Bacterial 16S rRNA gene as formerly employed [42], [45], [46], using the primers 104F (5′-GGCGVACGGGTGAGTAA-3′) and 530R (5′-CCGCNGCNGCTGGCAC-3′). Amplicon pyrosequencing was performed with 454 GS-FLX Titanium protocols [47]. We qualified raw sequences by trimming off low-quality bases and removing low-quality, chimeric sequences, and singletons by using the Quantitative Insights Into Microbial Ecology (QIIME) software package version 1.6.0 [48]. After quality control, aligning and clustering as described [49], we obtained the operating taxonomic units (OTUs) at 97% sequence similarity and assigned taxonomy to OTUs by using the Ribosomal Database Project classifier with a 50% confidence threshold [50]. We obtained the following number of qualified sequences: Cuzdrioara soil, 6,924; Carolina sediment, 7,904; and Parris Island sediment, 1,486; ZARA-10 enrichment culture, 3,141; LINA-09 enrichment culture, 2,847; and ISLA-08 enrichment culture, 2,205. The sequences reported in this paper were deposited in the Sequence Research Archive (SRA) at NCBI with the following accession numbers: SAMN02361223, SAMN02361224, SAMN02361225, SAMN02361226, SAMN02361227, and SAMN02361228.

To compare microbial diversity on an equal basis, we performed rarefaction on the OTU table by randomly selecting sequences at the same depth (1,486: the least number of sequences throughout samples) with a python script in QIIME. From the rarefied OTU table, we assessed microbial diversity within a sample (alpha diversity) through Phylogenetic Diversity (PD) using the PD Whole Tree estimator in QIIME. We also employed phylogeny-based UniFrac distance matrix [51] and generated principal coordinate analysis (PCoA) to evaluate similarity among soil and sediment samples and soil/sediment-free enrichment cultures.

We enumerated the 16S rRNA genes of *Dehalococcoides mccartyi* and *Archaea*, as well as *Dehalococcoides mccartyi* reductive dehalogenase genes, *tceA*, *vcrA*, and *bvcA* in the soil/sediment samples and the enrichment cultures *via* quantitative real-time PCR (qPCR). Triplicate TaqMan assays were setup for samples and standard curve points and contained the following per 10 µL reaction: 4.5 µL of 2.5×5 PRIME MasterMix (5 PRIME, Inc., USA), 0.5 µL F' and R' primers from 10 µM stocks, 0.03 µL probe from 100 µM stock, 0.47 µL PCR water, and 4 µL DNA template (diluted 1∶10). The primers and probes were previously described for *Dehalococcoides mccartyi* 16S rRNA gene, *tceA*, and *vcrA* [52]; *bvcA* [53]; and *Archaea* 16S rRNA gene [54], [55]. We used an Eppendorf Realplex 4S realcycler with a PCR temperature profile and cycles as outlined by Ziv-El et al. [25].

## Results and Discussion

### Biostimulation of Indigenous *Dehalococcoides* in Microcosms from Distinct Environments

**Figure 1:**
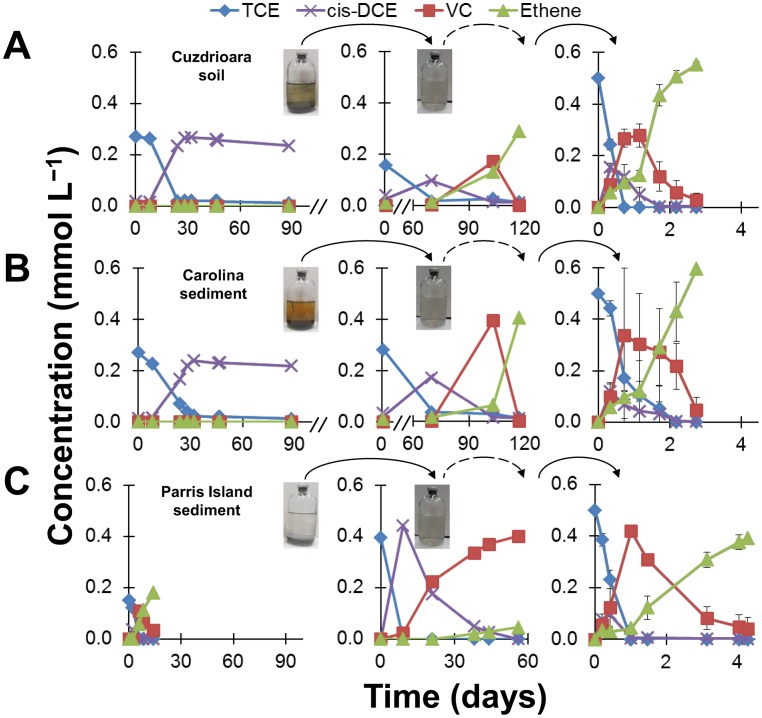
Biostimulation of chlorinated ethene-respiring communities containing *Dehalococcoides*. Dechlorination of TCE in microcosms (left panels), first transfers from microcosms (middle panels), and enriched soil/sediment-free cultures (right panels). The microcosms (left panels) were setup with (A) uncontaminated garden soil, (B) uncontaminated mangrove sediment, and (C) PCE-contaminated groundwater sediment. A total of 26 microcosms were established. (A)–(B) (left panels) Cuzdrioara and Carolina microcosm replicates exhibited the same pattern for reductive dechlorination product formation and one replicate is shown. Eight Parris Island replicate microcosms from different core depths formed VC and ethene within 30 days after microcosms were established. (C) (left panel) One representative VC and ethene-producing microcosm is presented. The dashed arrows represent an additional transfer not shown. The time-course experiments from the right panels (A–C) are from the third consecutive addition of 0.5 mmol L^−1^ TCE. The error bars in the right panels show standard deviation of triplicate cultures. Note the time scale differences between left, middle, and right panels.

We utilized uncontaminated and contaminated soil and sediments from three geographically-distinct environments to assess the biological potential for dechlorination of TCE and the subsequent enrichment of *Dehalococcoides* in a total of 26 microcosms. Figure 1 (left panels) shows the dechlorination profile in microcosms amended with TCE, lactate, and methanol. As depicted in Figure 1A–B (left panels), *cis*-DCE was the end product from TCE reduction in all microcosms set up with uncontaminated soil and sediment from Cuzdrioara and Carolina, respectively. Production of ethene from PCE reductive dechlorination was previously recorded in microcosms established with uncontaminated river sediments in Löffler et al. [56]. In the microcosms with Parris Island sediment, dechlorination proceeded beyond *cis*-DCE (Figure 1C, left panel). A representative Parris Island microcosm producing VC and ethene is shown in Figure 1C (left panel). In fact, 40% of the microcosms from different core depths formed VC and ethene as early as 30 days after microcosm establishment. The dechlorination activity in our laboratory microcosms (Figure 1C, left panel) is consistent with reports of indigenous dechlorinating activity at the Parris Island site, where TCE, *cis*-DCE, VC, and ethene were detected from dechlorination of PCE after biostimulation with emulsified vegetable oil [41].

We sought to understand the differences between the microcosms that could or could not be biostimulated to produce VC and ethene. qPCR data revealed that *Dehalococcoides mccartyi* were present in the three soil/sediment inocula. Interestingly, the 16S rRNA gene copies of *Dehalococcoides mccartyi* were lowest in the Parris Island sediment (Figure S1), even though Parris Island microcosms readily dechlorinated TCE to VC and ethene (Figure 1C, left panel). In Parris Island and Carolina inocula, *Dehalococcoides mccartyi* were detected in a range of 10^5^ *Dehalococcoides mccartyi* 16S rRNA genes g^−1^ sediment, while in Cuzdrioara they were detected at 10^6^ genes g^−1^ soil (Figure S1). The amount of *Dehalococcoides mccartyi* gene copies contained in our samples are comparable to previously published *Dehalococcoides*-like *Chloroflexi* densities from a variety of soils and sediments [57]. Note that the characterized reductive dehalogenase genes (*tceA*, *vcrA* and *bvcA*) coding for enzymes involving dechlorination of TCE to VC (TceA [58]), *cis*-DCE and VC to ethene (VcrA [59] and BvcA [60]) were also present in the three soil/sediment inocula (Figure S1). The presence of the characterized reductive dehalogenase genes confirmed that the microbiological potential of *Dehalococcoides mccartyi* was not the limitation and that overlapping function redundancy for complete reduction existed in the *cis*-DCE stalled microcosms and those producing ethene.

Lactate and methanol were readily fermented through acetate and propionate, and methane evolution was recorded within the first weeks of incubation in these microcosms (Figure S2). Hence, we postulated that the electrons from the fermentable substrates were being utilized by H_2_-competing microorganisms growing on electron acceptors from the soil and sediment and on HCO_3_^−^ from the medium. On day 46, we transferred soil/sediment-free supernatant from each microcosm bottle stalled at *cis*-DCE (Figure 1A–B, middle panels). The original Cuzdrioara and Carolina microcosms stalled at *cis*-DCE were biostimulated two additional times with 5 mM lactate (day 46 after the transfer and day 180) to replenish the electron donor, and were incubated for up to 200 days. It is likely that some H_2_ produced during fermentation was initially available to *Dehalococcoides* for dechlorination of TCE to *cis*-DCE. However, the additional electron donor and the extended incubation did not further advance *cis*-DCE reduction. This indicated that *Dehalococcoides* able to dechlorinate past *cis*-DCE were outcompeted by other microorganisms, and H_2_ became limiting for *Dehalococcoides* in the microcosms. On the other hand, methane concentrations increased throughout incubation, reaching up to 7.2 mmol L^−1^ and 8.8 mmol L^−1^ in Cuzdrioara and Carolina microcosms, respectively (Figure S2), showing unmistakably that methanogenesis was one of the biological processes benefiting from our biostimulation efforts. Contrary to the observations from Figure 1A–B (left panels) which show only partial reduction of TCE to *cis*-DCE, we *did* achieve complete dechlorination of TCE to ethene in the soil/sediment-free mixed cultures resulting from the microcosm transfers (Figure 1A–B, center and right panels). This confirmed that indigenous *cis*-DCE- and VC-respiring *Dehalococcoides* could indeed be biostimulated, and brings supporting evidence that the dilution of the soil/sediment and donor-competing microbial sinks facilitated this outcome.

### Characterization of Soil/Sediment-Free Cultures Enriched in *Dehalococcoides mccartyi*

Through Figure 1A–B (left panels), our study exemplifies that microcosm data can fail to predict the “true” reductive dechlorination potential in an environment. In spite of this, we showed that ethene could be obtained as the reduced end product of TCE dechlorination through a specific enrichment process. We further sought to evaluate the impact of selective enrichment and culturing on the soil/sediment-free microbial communities developed from the three distinct environments. ZARA-10, LINA-09, and ISLA-08 were maintained under identical batch-fed growth conditions. The majority of the electrons from lactate and methanol were channeled to fermentation products (acetate and propionate, Figure S3). HCO_3_^−^-reducing methanogens belonging to *Archaea* continued to be detected (Figure S1) and active (Figure S2) in all enrichment cultures; however, as shown in Figure S2, methane production diminished when compared to the activity in the microcosms. As for the TCE dechlorinating activity, Figure 1A–C (right panels) reveals that, as a result of the enrichment protocol, fast reduction of TCE to ethene was achieved in all three enrichment cultures, regardless of the environment where the microbial inocula originated. Moreover, the culture performance parameters tabulated in Table 2 show similarly high maximum observed transient conversion rates (on the order of mM Cl^−^ released per day) in ZARA-10, LINA-09, and ISLA-08 enrichment cultures, and dechlorination of 0.5 mmol L^−1^ TCE to ≥80% ethene in as short as 1.7 days.

**Table 2:** Characterization of *Dehalococcoides mccartyi*-containing cultures enriched in this study.

	Enrichment culture		
	ZARA-10	LINA-09	ISLA-08
**Highest transient conversion rate** a			
(mmol Cl^−^ released L^−1^ d^−1^)	2.7±0.34	2.4±0.43	2.5±0.08
**Conversion of 0.5 mmol L^−1^ TCE to ≥80% ethene** b			
(days)	1.7	2.8	4.0
**Conversion of 1 mmol L^−1^ TCE to ≥80% ethene** b			
(days)	2.3	5.9	8.8
**Final ** ***Dehalococcoides mccartyi*** ** densities** c			
(cells mL^−1^) at 0.5 mmol L^−1^ TCE	9.6×10^8^	2.2×10^9^	1.9×10^9^
(cells mL^−1^) at 1 mmol L^−1^ TCE	2.3×10^9^	1.8×10^9^	2.3×10^9^
**Yield ** ***Dehalococcoides mccartyi*** d			
(cells µmol^−1^ Cl^−^ released)	2.6×10^8^	1.8×10^8^	2.3×10^8^

a Rates calculated between two consecutive sampling points. The transient rates were highest for all cultures on the third addition of 1 mmol L^−1^ TCE.

b Conversion times reported from independent experiments for the third consecutive addition of TCE.

c Final densities after three consecutive additions of TCE.

d Yields were calculated from the change in the 16S rRNA gene copies measured by qPCR divided by the change in concentration of TCE reduced to ethene.

### Insights from the Composition of Microbial Communities

**Figure 2:**
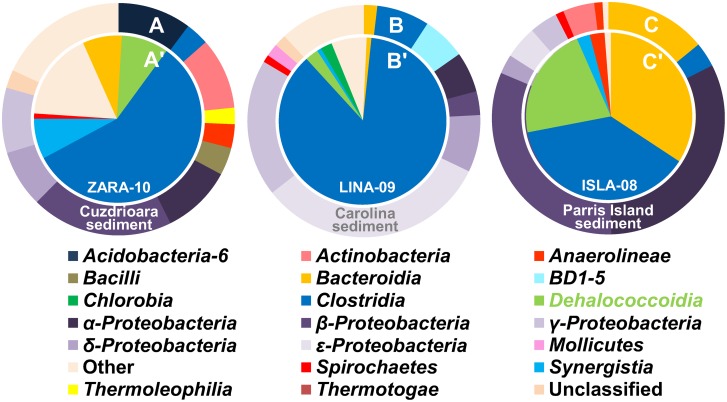
Bacterial composition at the class level as determined by 454 pyrosequencing of the V2-V3 region of the 16S rRNA gene. The outer pie charts (A–C) represent the relative abundance of select classes in the Cuzdrioara uncontaminated soil, (B) Carolina uncontaminated sediment, and (C) Parris Island contaminated sediment. The inner pie charts (A'–C') show the five most abundant classes in the respective soil/sediment-free enrichment cultures, ZARA-10, LINA-09, and ISLA-08. The classified taxa presented contributed to at least 1% of the total relative abundance and are organized in alphabetical order.

To gain further insights into the differences between the microcosms that could or could not be biostimulated beyond *cis*-DCE, we took advantage of high throughput sequencing. The outer pie charts in Figure 2A–C illustrate the 454 pyrosequencing data at class level from Cuzdrioara soil, Carolina sediment, and Parris Island sediment. The class of interest for TCE to ethene respiration is *Dehalococcoidia*, containing the genera *Dehalococcoides* [1], *Dehalogenimonas* [61], and *Dehalobium* [62]. *Dehalococcoidia* was in low abundance in all soil and sediment samples (<1% of total sequences: Cuzdrioara, 0.2%; Carolina, 0.25%; Parris Island, 0.4%), and hence, is not shown in Figure 2A–C. As seen in Figure 2A–C (outer pie charts), the Cuzdrioara and Carolina environments were more diverse than the Parris Island sediment and were predominantly populated by *α*-, *β*-, *γ*-, *δ*-, and *ε*-*Proteobacteria*. The microbial communities in Cuzdrioara soil and Carolina sediment (Figure 2A–B) concur with previously described microbial communities where sulfate [49], [63], sulfur [63], iron [64], nitrate [49], [65], and HCO_3_^−^ [42], [66] were abundant. Compared to Cuzdrioara and Carolina samples, the Parris Island sediment contained fewer classes (Figure 2C) and had a higher relative abundance of *Dehalococcoidia*.

While in low abundance in the soils and sediments, relative to the other bacterial classes, *Dehalococcoidia* became one of the most prevalent taxa in all three enrichment cultures. This is depicted in the inner pie charts in Figure 2A′–C′: ZARA-10, 9%; LINA-09, 3%; ISLA-08, 21%. Furthermore, the predominant classes in all three originating environments, *α*-, *β*-, *γ*-, and *ε*-*Proteobacteria* were absent (zero sequences) in the enrichment cultures (Figure 2A′–C′). We believe this outcome was obtained by removing or diluting other competing electron acceptors present in the soil/sediment which were supporting microbial guilds competing for the electron donor. The most abundant sequences for all three enrichment cultures belong to *Clostridia*. The obvious increase in relative abundance of this class containing fermenting bacteria was the result of feeding excess fermentable substrates (lactate and methanol) throughout the enrichment process.

The family *Geobacteraceae* within the *δ*-*Proteobacteria*, containing bacterial members known to respire TCE [67], was maintained in low abundance (<1%) in the enrichment cultures ZARA-10 and LINA-09. A different response was found for the enrichment of anode-respiring bacteria by Miceli et al., who employed the same soil and sediment samples from Cuzdrioara and Carolina in microbial electrochemical cells [64]. In the study by Miceli et al., when garden soil from Cuzdrioara was used as a microbial inoculum, the resulting enrichment was dominated by *δ*-*Proteobacteria* (∼90% relative abundance) [64]. When the source of microbes was Carolina mangrove sediment, ∼60% of the enriched biofilm was comprised of *α*-, *γ*-, and *δ*-*Proteobacteria* [64]. The elimination or minimization of these *Proteobacterial* classes in the enrichment cultures from our study is attributed to the selective conditions provided in the medium, with TCE and HCO_3_^−^ as sole electron acceptors.

### Diversity Analyses Reveal Convergent Enrichment Cultures

**Figure 3:**
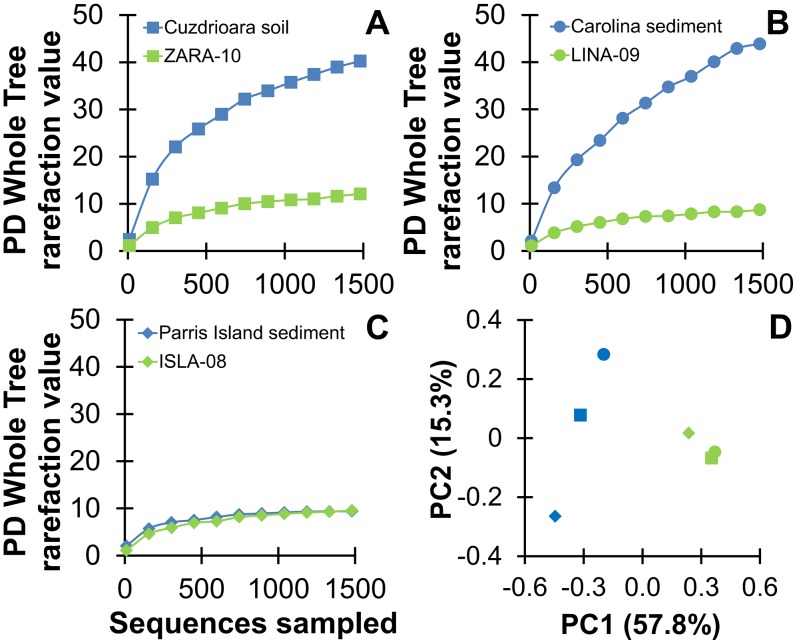
Alpha and beta microbial diversity analyses. (A)–(C) Rarefaction plots for PD Whole Tree measurements from the 454 analysis using trimmed, equal sequencing depth OTUs (1,486) per sample. (D) Weighted UNIFRAC distance calculated after trimming the samples to equal sequence depth in QIIME. The PCoA plot was generated by grouping the samples into two categories (soils/sediments vs. enrichment cultures). The color blue corresponds to the soil/sediment samples, while green corresponds to the soil/sediment-free enrichment cultures.

We conducted alpha diversity analyses using phylogeny-based metrics (PD index) to estimate microbial diversity. As depicted in Figure 3A–C, the PD values were low and comparable for all three enrichment cultures, indicating low microbial diversity. This was achieved from Cuzdrioara soil and the Carolina sediment (Figure 3A–B), which had PD values approximately four fold higher than that of Parris Island (Figure 3C). Furthermore, PCoA between samples (beta diversity) reveals that heterogeneous soil and sediment samples (blue symbols, Figure 3D) converged to very similar, highly efficient TCE-respiring microbial communities after the enrichment (green symbols, Figure 3D).

### Effect of Enrichment Techniques on Growth of *Dehalococcoides mccartyi*

**Figure 4:**
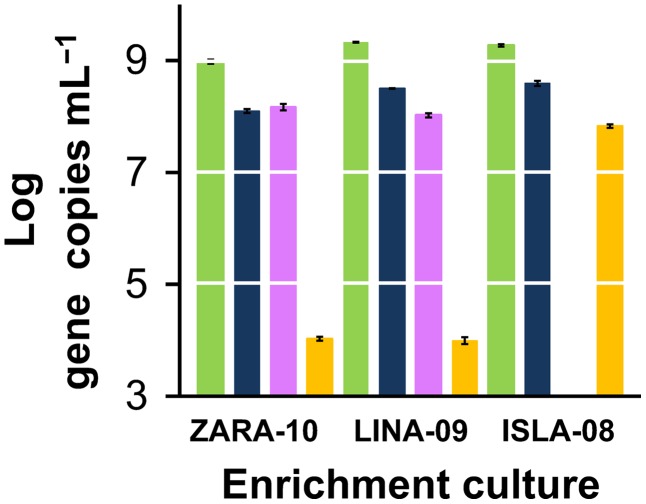
Enumeration of *Dehalococcoides mccartyi* in enrichment cultures. qPCR tracking *Dehalococcoides* 16S rRNA genes and their reductive dehalogenase genes, *tceA*, *vcrA*, and *bvcA* in the enrichment cultures after three consecutive additions of 0.5 mmol L^−1^ TCE. The plot is representative of triplicate cultures and the error bars show standard deviations of triplicate qPCR reactions.

Enumeration of *Dehalococcoides mccartyi* was achieved through qPCR targeting the 16S rRNA genes (Table 2 and Figure 4) and their reductive dehalogenase genes, *tceA*, *vcrA*, and *bvcA* (Figure 4). The three enrichment cultures exhibited some microbiological differences with respect to the *Dehalococcoides mccartyi* strains enriched. For example, *vrcA* was not detected in ISLA-08 (Figure 3) even though it was present in the Parris Island sediment (Figure S1). However, common to all enrichment cultures were the very high (and similar) densities of *Dehalococcoides mccartyi* of ∼10^9^ *Dehalococcoides* cells mL^−1^ (or 10^12^ cells L^−1^) (Table 2 and Figure 4). These densities were mainly the result of the enrichment protocol, where high doses of TCE (1.5–3 mmol L^−1^ total over three additions) were subsequently reduced by *Dehalococcoides mccartyi* coupling growth to dechlorination. The minimization of microbial competitors, especially hydrogenotrophic methanogens, also contributed to achieving these high *Dehalococcoides* densities. The high concentrations of TCE fed to *Dehalococcoides* may have also directly suppressed the methanogenic populations as initially reported by DiStefano et al. in enrichments fed with PCE, methanol, and yeast extract [15].

Taken together, the rates and the times required for complete reduction to ethene of the TCE supplied (Figure 1, right panels), and the resulting *Dehalococcoides mccartyi* concentrations (Table 2 and Figure 4) compare favorably to previously published values tabulated by Ziv-El et al. [25], [68]. Dechlorination rates and *Dehalococcoides mccartyi* densities, two important and interconnected factors for successful bioremediation, vary sometimes by orders of magnitude [25], [68] between previously characterized cultures. A contributing factor to these variances could be the enrichment technique employed, as the compared studies used different conditions and different stimulation techniques for the development and cultivation of reductively dechlorinating cultures. Our results support the idea that a robust microbial community, where *Dehalococcoides* thrive, can be achieved under the enrichment conditions described herein.

### Bioaugmentation of Microcosms with Their Respective Enrichment Cultures

**Figure 5:**
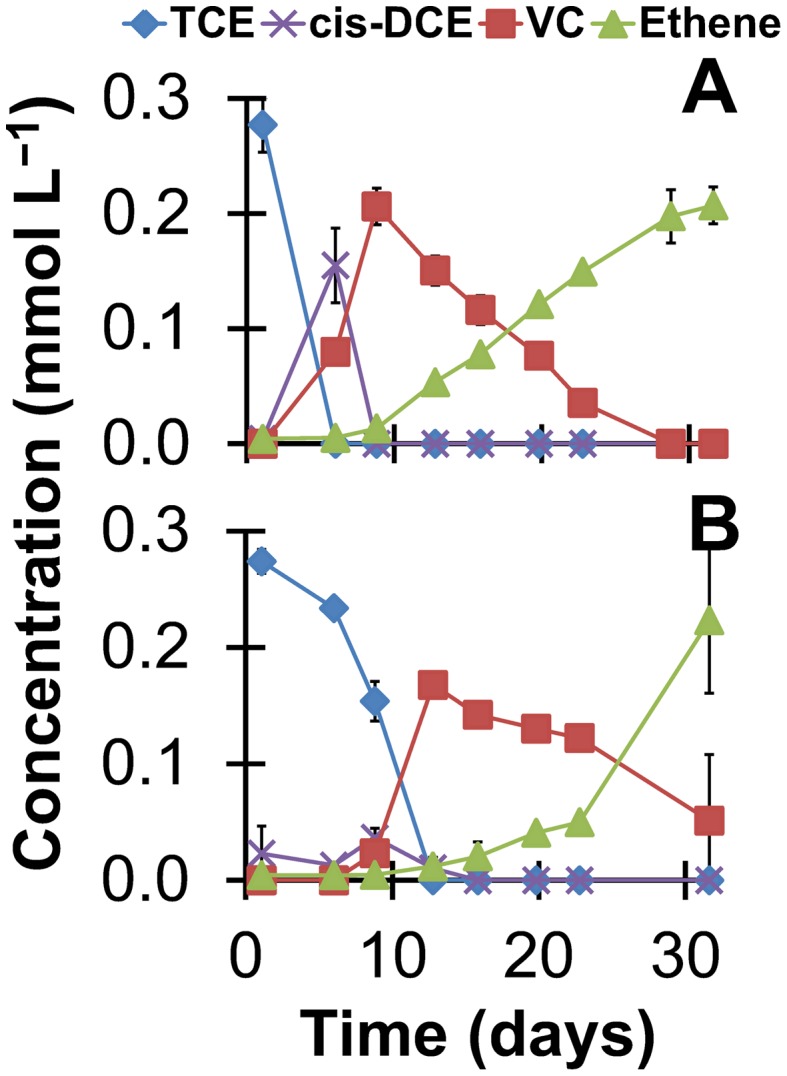
Bioaugmentation of microcosms with their respective enrichment cultures. Dechlorination of TCE in (A) in Cuzdrioara soil microcosms bioaugmentated with ZARA-10 enrichment culture and in (B) Carolina sediment microcosms bioaugmented with LINA-09 culture. The inoculum used for these experiments was 1% vol/vol.

One important aspect of our study, exemplified with the Cuzdrioara soil and Carolina sediment, is the ability to stimulate the production of VC and ethene and the growth of *cis*-DCE and VC-respiring *Dehalococcoides*. To strengthen this point, we designed a simple experiment to show that, once *Dehalococcoides* are enriched and in high abundance, they can better compete in the complex soil or sediment environments from which they originated. For this, we re-established microcosms containing Cuzdrioara soil and Carolina sediment and bioaugmented the microcosms with a 1% vol/vol inoculum of ZARA-10 or LINA-09 enrichment culture, respectively, to more appropriately reflect the dilution factor in bioremediation scenarios at contaminated sites. As seen in Figure 5A–B and supportive of our hypothesis, with both enrichment cultures dechlorination of TCE proceeded to ethene and we achieved close to complete dechlorination of 0.25 mmol L^−1^ TCE to ethene in ∼30 days. The rates of reductive dechlorination obtained with this smaller inoculum exclude the possibility that the *cis*-DCE stall resulted from components in the soil/sediment inhibiting the native *Dehalococcoides*.

### Outlook on Bioremediation Using *Dehalococcoides*

For bioremediation of PCE or TCE contaminated sites, microcosm experiments have historically been utilized as indicators of indigenous microbial activity [14]. The results of microcosm experiments help researchers and bioremediation practitioners decide whether biostimulation or bioaugmentation is the appropriate treatment for decontamination of environments polluted by chlorinated solvents [14]. In cases where incomplete dechlorination was observed in microcosms, this has been attributed to the presence of inhibitors in the soil or the lack of *Dehalococcoides mccartyi* capable of complete dechlorination. Our findings clearly show that neither result from Cuzdrioara nor Carolina biostimulated microcosms could be explained by these two hypotheses. Instead, an electron donor competition is proposed, supported by our data, in which components of the soil or sediment serve as electron acceptor for competing H_2_-oxidizing microorganisms.

Our results bring experimental evidence towards a new possible explanation to “unsuccessful” microcosm experiments. If indeed microbial competition for electron donor is a major determining factor in the success of established microcosms, it will certainly be a determining factor in bioremediation as well, and adding excess electron donor to biostimulate could prove unsuccessful. Furthermore, through the soil/sediment-free enrichment cultures developed, we bring evidence linking fast rates of TCE to ethene dechlorination and high densities of *Dehalococcoides mccartyi* to the selective enrichment protocol. In our study, these rates and densities were independent of the origin of the microbial inocula and the end-product of reductive dechlorination in microcosms, which bring about implications for potentially improving bioremediation in chlorinated ethene-contaminated environments.

## Supporting Information

Figure S1 **Enumeration of** ***Dehalococcoides mccartyi*** **in environmental inocula and enrichment culture inocula.** qPCR tracking *Dehalococcoides mccartyi* 16S rRNA gene, *tceA*, *vcrA*, and *bvcA* and *Archaeal* 16S rRNA gene. The filled bars represent the relative abundance of the targeted genes in the soil and sediment inocula (Cuzdrioara, Carolina, and Parris Island) before microcosm establishment. The empty bars show the target gene concentrations in the soil/sediment-free enrichment cultures used as inocula for the experiments shown in the right panels Figure 1A–C. The error bars are standard deviations of triplicate qPCR reactions.
(TIFF)

Figure S2 **Methane production in microcosms and subsequent enrichment cultures.** (A)–(B) Left panels: time-course methane measurements in Cuzdrioara and Carolina microcosms biostimulated with fermentable substrates. (A)–(B) Right panels: final methane concentrations recorded in Cuzdrioara and Carolina microcosms (end of experiments from Figure 1A–B, day 200) and final methane concentrations in ZARA-10 and LINA-09 enrichment cultures (end of experiments from Figure 1A–B, day 2.8).
(TIFF)

Figure S3 **Distribution of electrons from lactate and methanol in enrichment cultures.** Typical electron balance showing distribution of electrons from 5 mM lactate and 12 mM methanol to the main processes occurring in the soil/sediment-free cultures. This electron balance was generated from data collected from triplicate cultures after three consecutive additions of fermentable substrates and TCE.
(TIFF)
