# Supplementary figures and images for: Selective Enrichment Yields Robust Ethene-Producing Dechlorinating Cultures from Microcosms Stalled at cis-Dichloroethene
Source: PLoS One. 2014 Jun 20;9(6):e100654. doi: 10.1371/journal.pone.0100654 (PMC4065118; doi:10.1371/journal.pone.0100654)

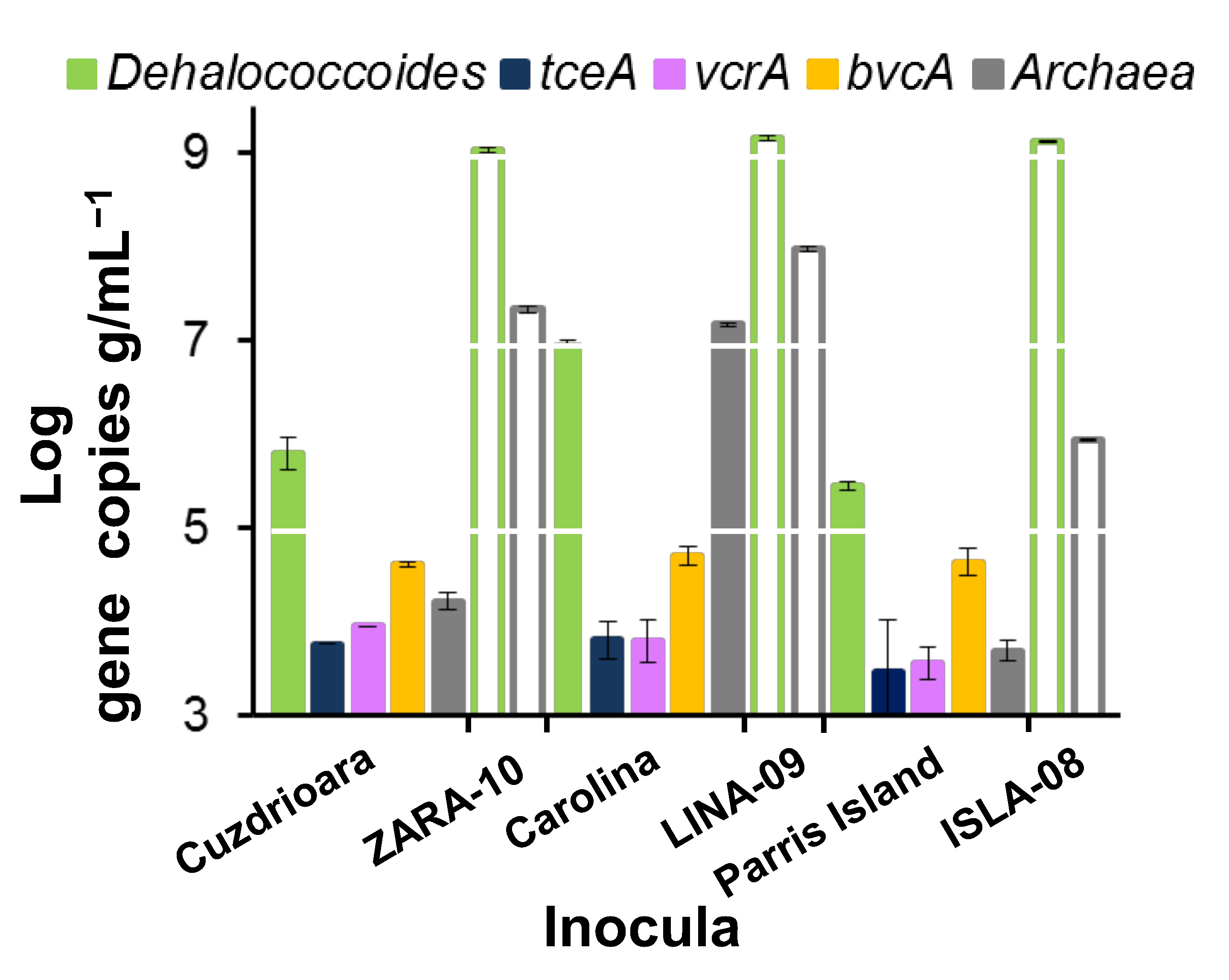

Supplement: Figure S1 — Enumeration of Dehalococcoides mccartyi in environmental inocula and enrichment culture inocula. qPCR tracking Dehalococcoides mccartyi 16S rRNA gene, tceA, vcrA, and bvcA and Archaeal 16S rRNA gene. The filled bars represent the relative abundance of the targeted genes in the soil and sediment inocula (Cuzdrioara, Carolina, and Parris Island) before microcosm establishment. The empty bars show the target gene concentrations in the soil/sediment-free enrichment cultures used as inocula for the experiments shown in the right panels Figure 1A–C. The error bars are standard deviations of triplicate qPCR reactions. (TIFF) [file pone.0100654.s001.tif]

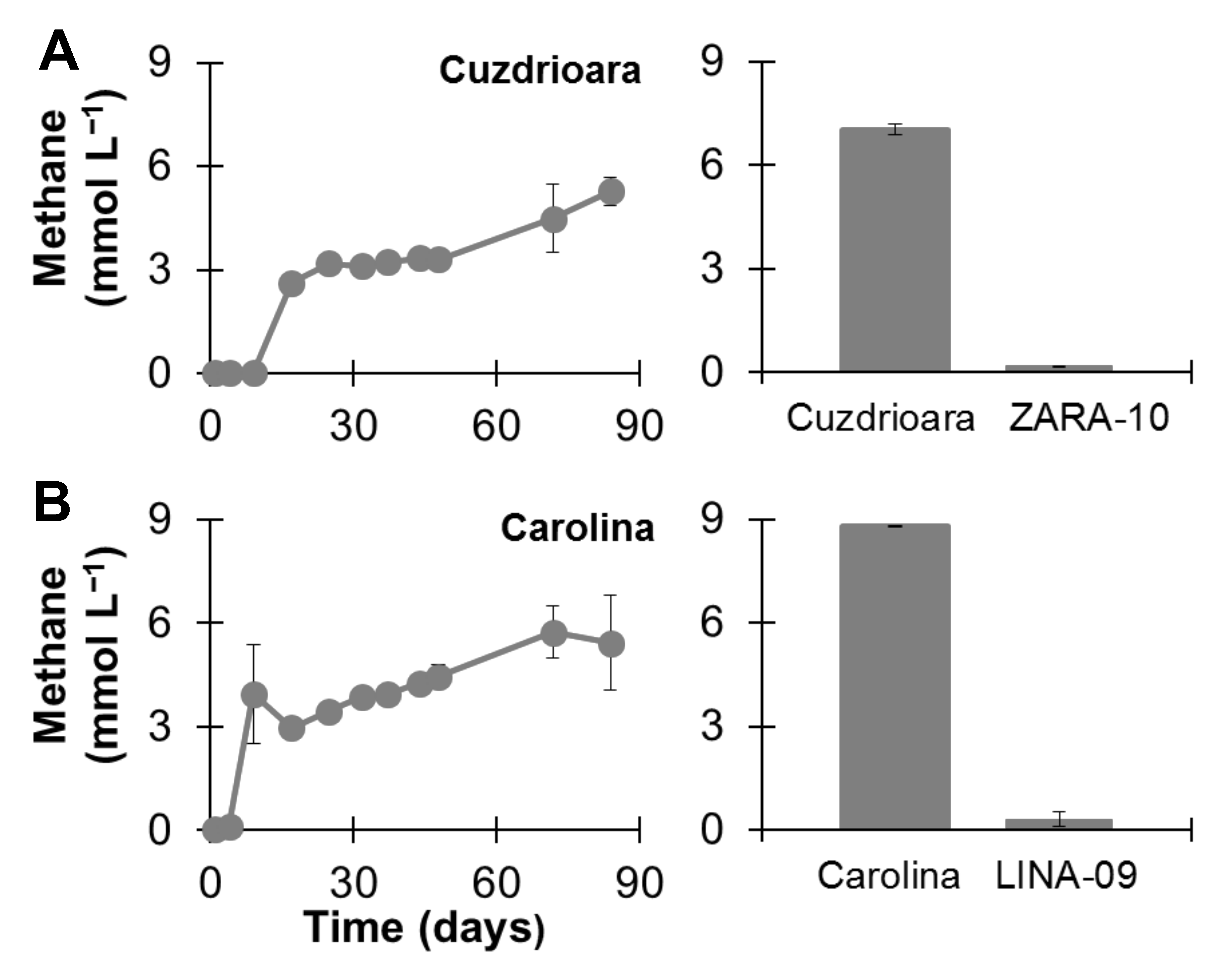

Supplement: Figure S2 — Methane production in microcosms and subsequent enrichment cultures. (A)–(B) Left panels: time-course methane measurements in Cuzdrioara and Carolina microcosms biostimulated with fermentable substrates. (A)–(B) Right panels: final methane concentrations recorded in Cuzdrioara and Carolina microcosms (end of experiments from Figure 1A–B, day 200) and final methane concentrations in ZARA-10 and LINA-09 enrichment cultures (end of experiments from Figure 1A–B, day 2.8). (TIFF) [file pone.0100654.s002.tif]

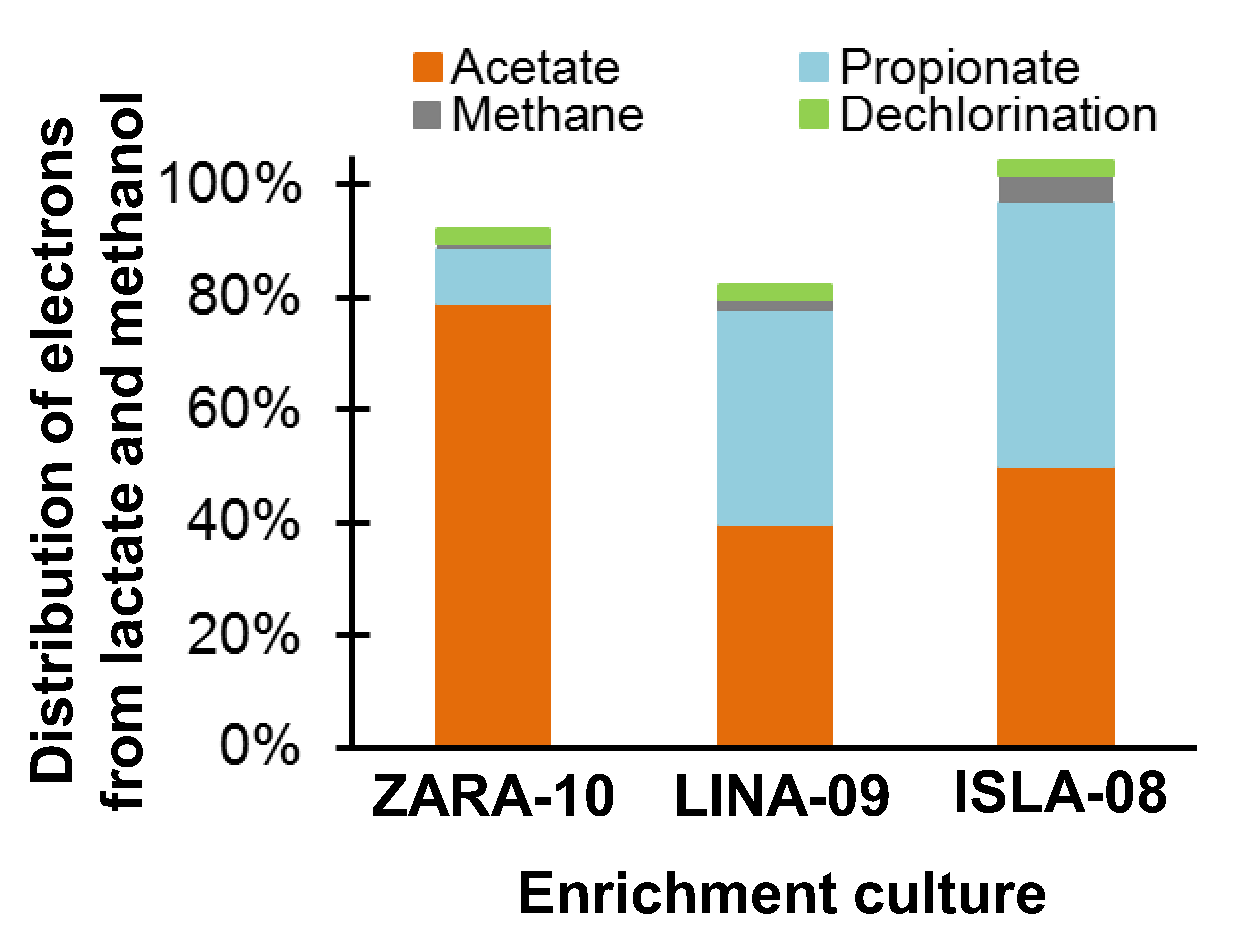

Supplement: Figure S3 — Distribution of electrons from lactate and methanol in enrichment cultures. Typical electron balance showing distribution of electrons from 5 mM lactate and 12 mM methanol to the main processes occurring in the soil/sediment-free cultures. This electron balance was generated from data collected from triplicate cultures after three consecutive additions of fermentable substrates and TCE. (TIFF) [file pone.0100654.s003.tif]
